# General Unified Microbiome Profiling Pipeline (GUMPP) for Large Scale, Streamlined and Reproducible Analysis of Bacterial 16S rRNA Data to Predicted Microbial Metagenomes, Enzymatic Reactions and Metabolic Pathways

**DOI:** 10.3390/metabo11060336

**Published:** 2021-05-24

**Authors:** Boštjan Murovec, Leon Deutsch, Blaž Stres

**Affiliations:** 1 Faculty of Electrical Engineering, University of Ljubljana, Tržaška 25, SI-1000 Ljubljana, Slovenia; bostjan.murovec@fe.uni-lj.si; 2 Biotechnical Faculty, University of Ljubljana, Jamnikarjeva 101, SI-1000 Ljubljana, Slovenia; leon.deutsch@bf.uni-lj.si; 3 Faculty of Civil and Geodetic Engineering, University of Ljubljana, Jamova 2, SI-1000 Ljubljana, Slovenia; 4 Department of Automation, Jožef Stefan Institute, Biocybernetics and Robotics, Jamova 39, SI-1000 Ljubljana, Slovenia; 5 Department of Microbiology, University of Innsbruck, Technikerstrasse 25d, A-6020 Innsbruck, Austria

**Keywords:** 16S rRNA, amplicon, Mothur, PICRUSt 2, Piphillin, genus, OTU, ASV, predicted metagenomes, predicted enzymatic reactions, predicted metabolic pathways, reproducible analyses, human microbiome, gut, intestine, mice

## Abstract

General Unified Microbiome Profiling Pipeline (GUMPP) was developed for large scale, streamlined and reproducible analysis of bacterial 16S rRNA data and prediction of microbial metagenomes, enzymatic reactions and metabolic pathways from amplicon data. GUMPP workflow introduces reproducible data analyses at each of the three levels of resolution (genus; operational taxonomic units (OTUs); amplicon sequence variants (ASVs)). The ability to support reproducible analyses enables production of datasets that ultimately identify the biochemical pathways characteristic of disease pathology. These datasets coupled to biostatistics and mathematical approaches of machine learning can play a significant role in extraction of truly significant and meaningful information from a wide set of 16S rRNA datasets. The adoption of GUMPP in the gut-microbiota related research enables focusing on the generation of novel biomarkers that can lead to the development of mechanistic hypotheses applicable to the development of novel therapies in personalized medicine.

## 1. Introduction

The gut microbiota is composed of a huge number of different bacteria, archaea, fungi and protozoa, next to viruses and various mobile elements [1,2]. All these microbes interact with the host, environmental stimuli and each other, thus producing an enormous diversity of chemical compounds that play a key role in host development, wellbeing and aging [3,4,5,6,7]. The advent of large scale microbiome studies generates analytical opportunities to understand how these communities operate and respond to their complex environmental stimuli [8]. Although knowledge of taxonomy and functional genes of microorganisms are both important, functional genes are more directly related to enzymatic reactions and metabolic pathways. It is increasingly recognized that the microbiome influences the host health state and disease progression. For instance, disease progression can range from mild gastrointestinal symptoms to inflammatory bowel disease and colorectal and liver cancer [9]. In addition, a range of diseases have been implicated in metabolic imbalances, ranging from metabolic syndrome and obesity to autoimmune diseases, psychological disorders and infections [9].

Amplicon sequencing of 16S rRNA has served as the key approach of the last decade for the understanding microbial community structure, dynamics and how organisms might influence or be influenced by environmental conditions [10]. Extensive sequencing of bacterial communities is generating large collections of datasets available through public repositories such as European Bioinformatics Institute (https://www.ebi.ac.uk/ accessed on 30 April 2021), CuratedMetagenomicsData [11], or individual studies [12]. These data have so far been described on the level of 16S rRNA taxonomy utilizing either (i) genus [12], (ii) 97–98.5% 16S rRNA identity operational taxonomic units (OTU) [13] or (iii) amplicon sequence variants (ASV) [14,15]. However, the processing and analyses of such datasets are highly diverse due to the high number of published and benchmarked pieces of software [16,17,18,19,20] and reports that lack significant technical details despite the Human Microbiome Project outlines and introduction of standard operating procedures [21,22,23].

In addition, this wealth of 16S rRNA data gives access to an untapped pool of information beyond the 16S rRNA taxonomy (genus, OTU, ASV), such as predicted functional genes, enzymatic reactions and metabolic pathways (Figure S1). The tools such as MicrobiomeAnalyst [24,25], PICRUSt [26], PICRUSt2 [27], Tax4Fun [28]; Tax4Fun2 [29] and Piphillin [30,31] link 16S rRNA sequence information to representative genome sequences and approximate metagenomics functional gene content relevant for the interpretation of the studied human disease phenomena and clinical metadata [32]. As a number of unexplored and large datasets encompassing thousands of samples and corresponding metadata are made available in repositories (e.g., [12,33] the analyses (genus, OTU, ASV) and improved predicted metagenomic, enzymatic and metabolic pathway datasets have the potential to unravel important taxonomic, functional, biochemical and metabolic findings (Figure S1).

However, in order to accomplish such intensive large scale data analyses effective workflows are required. These workflows should ideally (i) integrate various pieces software, (ii) streamline input and output formats, (iii) accommodate large datasets, (iv) maintain portability between benchtop PC and high performance computing clusters (HPC), (v) enable flexible (customizable) but also reproducible analyses (setting documentation) that can be (vi) shared with and utilized by other interested researchers.

**Figure 1:**
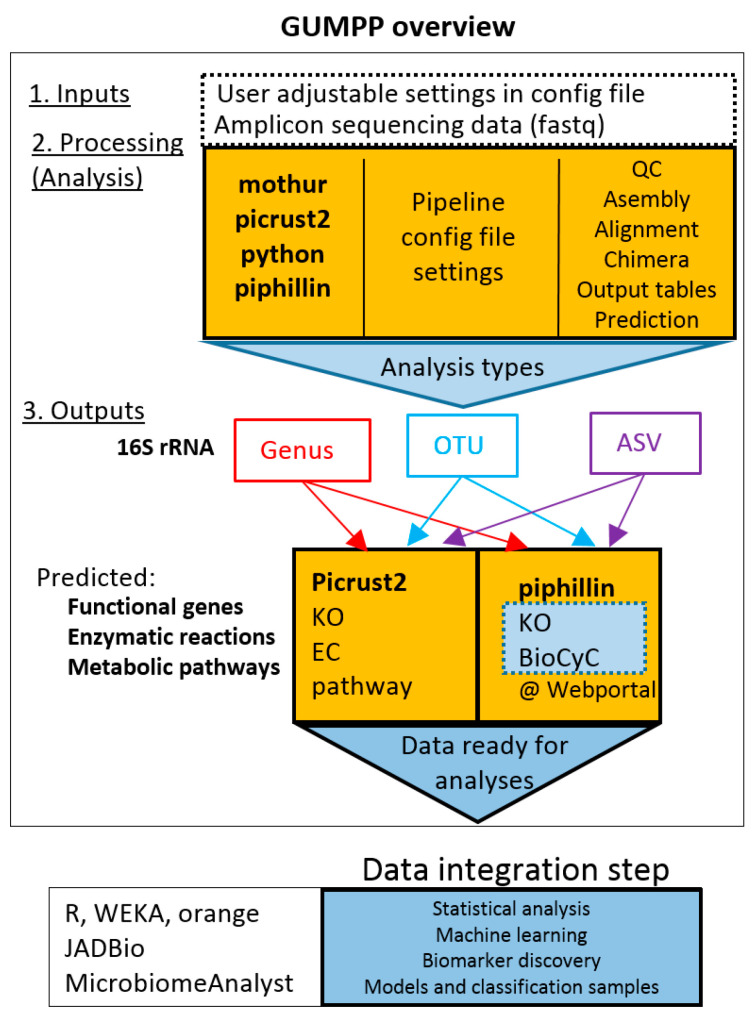
Schematic representation of the General Universal Microbiome Profiling Pipeline (GUMPP). The integral part consists of Mothur, PICRUSt2 and Piphillin outputs. Paired-end or single-end fastq sequence are used as input for mothur processing. The resulting biom and fasta files serve as an input for PICRUSt2. The data can be analyzed at genus-, OTU- and ASV- levels. QC–sequence quality control; OTU-Operational Taxonomic Units (generally 97% identity of 16S rRNA); ASV–Amplicon Sequence Variants (unique sequence variants). KO–KEGG Orthologs (Kyoto Encyclopedia of Genes and Genomes); EC-Enzyme Commission number; BioCyc-BioCyc collection of Pathway/Genome Databases. For each level, four output tables are generated (Please see Figures S1 and S2 for additional information). The resulting data can be analyzed in the data integration step using a variety of distinct machine learning approaches.

In this study, we introduce a workflow (Figure 1) that integrates Human Microbiome Project tested procedures for amplicon sequence analysis with one of the most popular programs Mothur [34], and PICRUSt2 [27] for prediction of metagenomic functional genes, enzymatic reactions and metabolic pathways. In addition, the workflow presented here generates also formatted inputs for Piphillin [30,31], another popular sister program for metagenomic predictions. The benchmarking of the integrated programs such as Mothur, PICRUSt2, Piphillin and other comparable sister programs were already reported before in numerous studies [16,17,18,19,20,23,27,30,31]. The inbuilt Human Microbiome Project standard operating procedures can be tailored according to user analytical preferences and sequencing details. The whole workflow is delivered as portable all-inclusive container (Singularity [35]; https://sylabs.io accessed on 14 April 2021) amenable for teaching or/and research purposes, using personal computer or HPC. Depending on the size of data and complexity of analyses (genus-, OTU-, ASV- levels), the GUMPP workflow enables maximum utilization of information present in the original 16S rRNA amplicon datasets by producing additional three data types approaching multiomics view of the microbiome: metagenomics functional genes, enzymatic reactions and metabolic pathways. All four data types can serve as inputs for machine learning to unravel novel mechanistic insight into human disease development in relation to microbiome characteristics. To showcase the efficient analyses and utilization of computing resources two datasets describing human (*n* = 307) and mice gut (*n* = 365) were used for demonstration purposes.

## 2. Results and Discussion

### 2.1. Design of GUMPP Workflow

GUMPP (http://gumpp.fe.uni-lj.si, accessed on 24 May 2021) is a freely available skeleton application for executing Mothur [34] using paired-end fastq files and executing the PICRUSt2 analyses next to producing also Piphillin [30,31] web-server input files (Figure 1). A single GUMPP run can process an arbitrary number of input files. Inputs are preprocessed by an integrated Mothur (V1.44.1) script in conjunction with Silva database (version 138), and creates biom and fasta representative sequence files as input for PICRUSt2 and outputs necessary for Piphillin [30,31]. The workflow was designed to support three levels of analysis differing in the increased extent of utilized information and fairness in data treatment: genus-, OTU- and ASV- levels (Figures S1 and S2). Users may freely replace the built in scripts and databases with their own. Customization of the built-in script (http://gumpp.fe.uni-lj.si) is also possible by template parameters.

**Figure 2:**
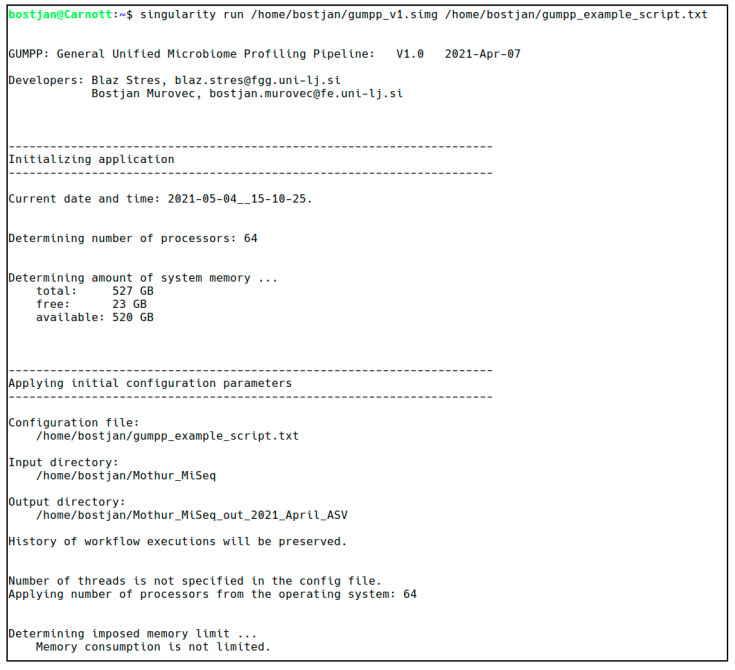
An example of the program startup and the initial checkups done by the Python code.

The primary design goal of the GUMPP application was to deliver efficient analyses and utilization of computing resources. The application relies on recently developed Singularity container technology ([35]; https://sylabs.io accessed on 14 April 2021) making the pipeline straightforward to use as all its ingredients are fully integrated, preinstalled and preconfigured in a ready-made Singularity image. These consist of the Mothur and PICRUSt2 programs, the needed Mothur scripts, two Silva taxonomy databases (V138 and V138 seed), a few supporting utilities written in C++, as well as a skeleton framework consisting of slightly less than 11,000 lines of Python code which orchestrates the execution of individual pieces and takes care of executing programs and building their command lines. The actual parameters under which the workflow is executed are at the control of the user (Figure 2, ESM Figures R1–R3).

Aside from reproducible execution of the workflow and the control of algorithm settings, GUMPP offers some additional benefits. First, results of Mothur preprocessing may optionally be stored in a specially crafted storage area, where each result is associated with its full context (hash of input files, Mothur script and its values of template parameters, or other relevant information). This enables efficient workflow re-executions with different Mothur and PICRUSt2 parameters. When GUMPP detects that upon its re-execution only PICRUSt2 parameters are changed, it instantly recycles the previously obtained Mothur results. This opens up a possibility of efficient experimenting with changed PICRUSt2 parameters to observe their impact on end results. In addition, Mothur processing is split into a common and an analysis specific part. If only analysis type or its related parameters are changed, the previously computed common results are again recycled instantly, which is a significant time saver, since the common part consists of e.g., sequence alignment to a taxonomy database. The system also enables crash recovery: in the case of GUMPP interruption during e.g., PICRUSt2 step (operating system crash, power outage, abort due to administrative policies on High-Performance Computer (HPC), upon restart only the PICRUSt2 step is re-executed. Crash recovery is completely automatic and transparent. A user need not to specify any directives to inform GUMPP that execution is being repeated.

The system is suitable for autonomous execution on domestic hardware as well as on HPC facilities. All command-line parameters and intermediate file formats are handled automatically by the system, enabling the experienced users to prescribe their own parameters for PICRUSt2 or for template Mothur script parameters in order to finetune the workflow execution.

In order to aid in documenting analyses and inspection of execution, GUMPP stores an accurate verbatim copy of its screen output as a part of end report. Also, the actual command lines, standard output streams, standard error streams and exit codes of individual programs are stored on a disk in a hierarchical way for easy navigation, inspection and debugging. Analysis setup relies on configuration files, where a complete workflow configuration is prescribed and hence also documented. GUMPP presented in this study thus builds on the highly popular and tested programs that were benchmarked in numerous past studies as reported before [16,17,18,19,20,27,30,31].

### 2.2. Reanalysis and Extension of Mice Gut Microbiome Data Using GUMPP: The Choice of Level of Analysis (Genus, OTU, ASV) Is far from Arbitrary

**Figure 3:**
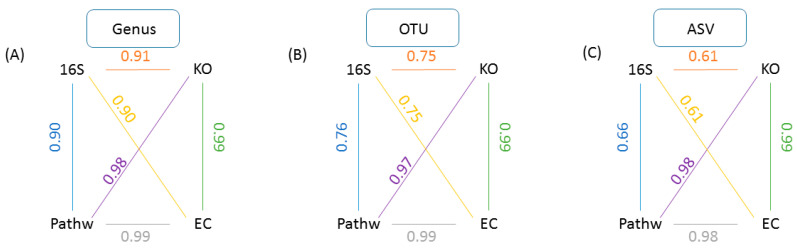
A within level analysis for all derived data types. A schematic representation of GUMPP generated data types analyzed at each of the three levels of 16S rRNA analysis (**A**) genus, (**B**) OTU, (**C**) ASV for the same sequence dataset and extended further to respective predicted functional genes (KO), enzymatic reactions (EC) and metabolic pathways (Pathw). Numbers designate the Mantel test correlation coefficients between various pairs of data types: (i) 16S and functional genes (KO)(orange), (ii) 16S and enzymatic reactions (EC) (yellow), (iii) 16S and metabolic pathways (Pathw) (blue), (iv) pathw and EC (pur-ple), (v) pathw and EC (grey), (vi) KO and EC (green). All analyses were performed with 9999 permutations and were statistically significant (*p* = 0.0001).

Mice data analysis using GUMPP enabled us to explore a technical question of how user reports on different taxonomic levels (genus; OTU; ASV) affected the exact relationships between underlying samples when studied utilizing the four data types (16S rRNA; functional genes; enzyme reactions; metabolic pathways). The results of Mantel test between taxonomic levels (Figure 3) show that the correlations between 16S rRNA vs. KO, 16S rRNA vs. EC and 16S rRNA vs. pathways decreased from 0.90, 0.91 and 0.90 at genus level, to 0.75, 0.75 and 0.76 at OTU level, and to 0.61, 0.61 and 0.66 at ASV level, respectively (*n* = 9999 permutations, *p* < 0.0002). The fact that ASV type of analysis resulted in lower correlations between datatypes is in line with past observations that there is little congruency between rather variable taxonomic descriptions of microbial communities and their corresponding even more diverse metagenomic functional gene makeup [36].

**Figure 4:**
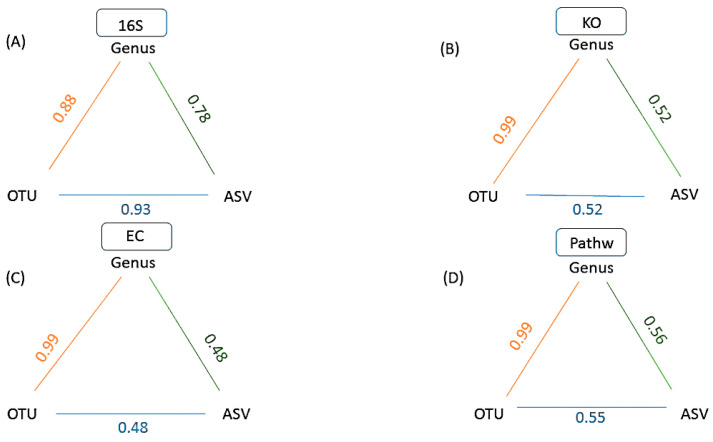
A schematic representation of GUMPP results showing a between level correlations for each data type: (**A**)16S rRNA (16S), (**B**) functional genes (KO), (**C**) enzymatic reactions (EC) and (**D**) metabolic pathways (Pathw). Numbers designate the Mantel test correlation coefficients between various pairs of levels for the same data type: (i) Genus and OTU (orange), (ii) OTU and ASV (blue), (iii) Genus and ASV (green). All analyses were performed with 9999 permutations and were statistically significant (*p* = 0.0001).

A between level analysis for each data type separately (Figure 4) illustrates the relationships between data of the same type, obtained using a different taxonomic level of analysis (Genus, OTU or ASV). The correlations > 0.88, describing the relationships between samples were retained only for distance matrices from genus and OTU levels of analyses and were also reproduced in all four data types (Figure 4). On the other hand, the initially high correlation between OTU and ASV at 16S rRNA level dropped below 0.55 for KO, EC and Pathway datasets, reflecting the increased number of categories (genus = 148, OUT = 1328, ASV = 13,244) and their different numerical abundance [11]. These results illustrate how the user selected levels of taxonomic assignment of the sequence data can affect the relationships between samples. Switching from genus level to utilizing ASV level of analysis does not only represent a way to maximize information content of the underlying 16S rRNA sequences [30], but it also represents a distorting transformation of the information due to the many predominantly biological limitations of such analyses: (i) differences in 16S rRNA gene copy numbers range from 1 to 15 in bacteria and 1 to 5 in Archaea [37], hence a frequently recovered sequence may represent a high copy number taxon of lesser abundance, or a low copy number taxon of higher abundance. This 16S copy number of the organism that contributed the sequence is estimated and data adjusted accordingly by utilizing PICRUSt2 [27] in GUMPP; (ii) intragenomic heterogeneity of 16S rRNA operons can be as large as 20.4%. Genus level classification encompasses rather divergent sequences of that specific genus into one category. On the other hand, single nucleotide polymorphism present within e.g., 10 copies of 16S rRNA operon within one organism represent distinct ASVs. In comparison to genus level analysis 16S rRNA variants of one organism are split to several ASV categories inflating ASV estimates of microbial taxonomic diversity and of functional diversity of underlying metagenomes [38,39,40]; (iii) In contrast, almost identical 16S rRNA copies and hence the lack of differences found within some genera do not enable stratification of species and strains present within, falsely deflating the number of present ASVs [10,38,39,40,41,42]; (iv) different hypervariable regions of 16S rRNA utilized in amplicon sequencing can result in additional distortion of signal relative to each other [43] hence compromising direct comparison of the results between studies utilizing distinct primers.

These cautionary notes listed above are intended to raise the awareness of the biological caveats of the genus, OUT and ASV levels of analyses for users. From this integrative view of biological influences the genus level analysis fits a more reserved type of analysis with arguably lower resolution, but congruent with an existing microbial taxonomy system in comparison to the ASV level of analysis, whereas OTU represents a compromise [14,44]. By utilizing ASV some genera expand into species and strains that have sufficient diversity within the 16S rRNA and contribute to ASVs, while other genera that contain species and strains with identical 16S rRNA in the region analyzed do not [14,44]. This biological distinction between genus, OTU or ASV levels of analysis has potentially large implications for the information forwarded to subsequent data types (functional genes, enzymatic reactions, metabolic pathays) irrespective of program utilized (PICRUSt, Tax4Fun, Piphillin or GUMPP).

Recent research highlights the risk of splitting a single bacterial genome into separate clusters when ASVs are used to analyze 16S rRNA gene sequence data. Although there is also a risk of clustering ASVs from different species into the same OTU when using broad distance thresholds, those risks are of less concern than artificially splitting a genome into separate ASVs and OTUs [14,44]. Based on the results presented here (Figure 3 and Figure 4), the choice of level of analysis (genus, OTU, ASV) is far from arbitrary and may lead researchers to draw different biological conclusions. The work presented in this study highlights the utility of GUMPP that enables researchers to analyze the data at all three levels at the same time, generates functional gene, enzymatic reactions and metabolic pathways datasets for downstream machine learning exploration in relation to human diseases [44].

### 2.3. Reanalysis and Extension of Human Gut Microbiome Data Using GUMPP

In this study a reanalysis of published human gut data (*n* = 307) [45] was conducted utilizing GUMPP at the levels of 16S rRNA, predicted metagenomes, enzymatic reactions and metabolic pathways. Differences between the gastrointestinal patients (*n* = 121) from a single ward and 186 healthy volunteers were explored. This effectively enabled us to reproduce previously reported findings [45] utilizing GUMPP. Analyses were extended to three additional data types: predicted functional genes, enzymatic reactions and metabolic pathways. First, as reported before in the original study [45], gut microbial community description was not sufficient to differentiate the subjects based on their underlying five broad medical diagnoses: (i) ulcerative colitis; (ii) Crohn’s disease, (iii) tumor (pancreatic, gastric or liver cancer), (iv) infection (pneumonia, cholangitis, hepatitis, gastritis or pancreatitis) and (v) other (cirrhosis or peptic ulcers, unidentifiable abdominal pain) [45]. The three mixed clusters independent of the underlying medical diagnosis were also reproduced (Figure S3), showing the robustness of GUMPP analysis. Second, by calculating the statistical power for each medical diagnosis a much larger number of samples (within each medical diagnosis) would be needed (*n* > 1000) to be able to build classification models for each diagnosis (Table S2). Third, the PCA representation confirmed the existence of a core microbiome in healthy individuals as described in the original study [45]. Human gut microbiome in patients was disturbed and significantly altered relative to the healthy microbiome (Figure S3).

Extending the original 16S rRNA analysis by GUMPP derived datasets (functional genes (KO), enzymatic reactions (EC), metabolic pathways (pathway)) enabled us to explore the differences between the gastrointestinal patients and healthy volunteers utilizing machine learning. This coupling between GUMPP produced datasets and machine learning enabled us to generate, train and validate four separate models for classification of samples (Figure S3; Supplementary Electronic Material) using JADBIO AutoML approach [46,47]. In short, at all four data levels, logistic ridge regression with penalty hyperparameter lambda = 0.1 was selected as the best interpretable model with AUC metrics of 0.937 (16S rRNA), 0.949 (KO), 0.954 (EC), and 0.947 (pathway) (Figure S3). For the best microbial feature selection, LASSO algorithm was selected for the most differentiating pathways, and Test-Budgeted Statistically Equivalent Signature (SES) algorithm was selected for the search of the most differentiating 16S rRNA, KO and EC between groups of patients and healthy individuals. Models based on KO and EC data performed better than those based on 16S rRNA and pathway data (Figure S4).

**Figure 5:**
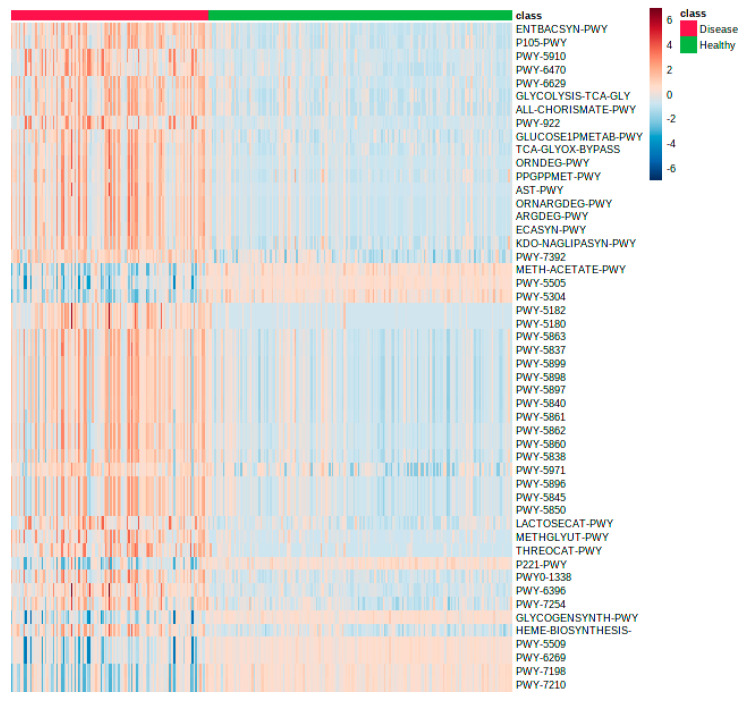
Heatmap showing the differences between the gastrointestinal patients (*n* = 121; red) from a single ward compared to 186 healthy volunteers (green) utilizing metabolic pathway information produced by GUMPP workflow from 16S rRNA data published before [45,59]. The first 50 most informative pathways are shown.

The optimization of model selection allowed us to reliably identify microbial features (taxa, functional genes, enzymatic reactions, metabolic pathways) from datasets analyzed and produced by GUMPP (Figure 1, Figures S2 and S3) that discriminated between gut microbiomes of gastrointestinal patients and healthy volunteers: 25 taxonomy level 16S rRNA OTUs, four KOs, 12 ECs and 15 pathways (Table S1). As the complete in-depth biological description of these results is beyond the scope of this study, the major differences between the healthy in diseased groups at the level of metabolic pathways are reported (Figure 5). The following findings are highlighted as proof of concept of GUMPP extended data analysis: lactocepin (EC:3.4.21.96; K01361) was identified in this study as one of the most important features at the level of functional genes and enzymatic reactions distinguishing healthy from IBD, UC and CD. High lactocepin in healthy cohort is involved in the selective degradation of pro-inflammatory chemokines, leading to reduced cell infiltration and reduced inflammation in IBD models [48,49]. Further, Cu+-exporting ATPase were also found to be significantly increased in healthy, hence acting at the level of enzymatic reactions in metabolism [50]. In contrast, the elevated values of the P-type Mg^2+^ transporter observed in gastrointestinal patients were previously shown to be important for increased virulence in *Escherichia coli* and *Salmonella thyphimurium* [51]. Similarly, higher activity of enzyme maltose-6’-phosphate glucosidase were identified in the maltose degradation pathway of *Enterococcus faecalis* leading to increased virulence of this pathogen [52]. Another important enzyme NADH oxidase that exerts the main protection against oxidative stress in the human gut was low in the healthy group [53]. Thiazole component of thiamine diphosphate biosynthesis pathway I and thiamine phosphate synthase were identified as important for separation between healthy and diseased individuals [54,55,56]. One of the distinguishing features was also the peptidoglycan biosynthesis pathway IV, previously described in *Ruminococcus gravus*, which is abundant in the intestines of patients with Crohn’s disease [57]. Bifibacterium shunt was identified as another pathway that has been previously shown to be important in providing positive health benefits to their host with its metabolic activities [58].

These results illustrate the insight supported by GUMPP into the potential differences in the gut microbiomes, functional genes, enzymatic reactions and metabolic pathways between the diffuse group of gastrointestinal patients (five medical diagnoses) and healthy cohort coupled to machine learning.

## 3. Materials and Methods

### 3.1. GUMPP Implementation

GUMPP utilization is described in user manual, Electronic Supplementary Materials, Config file, all available as part of this publication at http://gumpp.fe.uni-lj.si. Analyses running GUMPP were executed on a Dual Xeon system with 32 CPU cores (64 hyperthreads), 512 GB of RAM and 6 TB SATA disk. The runtime depends on the data size, sequencing depth and type of analysis (genus-, OTU-, ASV- level). For instance, human gut microbiome data analysis consisted of 307 samples, that each contained independent forward (R1) and reverse (R2) files. In total, it took <10 h, <50 h and <60 h runtime to finalize genus-, OTU- and ASV- levels of analyses, respectively. Similarly, runtime of analyzing less deeply sequenced mice dataset (*n* = 365 paired-end samples) took <4, <16 and <18 h to finalize genus-, OTU- and ASV- levels of analyses, respectively. Portability and HPC performance of the GUMPP generated in this study was confirmed on Leo3e (https://www.uibk.ac.at/zid/systeme/hpc-systeme/leo3e/ accessed on 30 April 2021) and Leo4 (https://www.uibk.ac.at/zid/systeme/hpc-systeme/leo4/ accessed on 30 April 2021) HPC infrastructure of the University of Innsbruck as described recently [60].

### 3.2. Sequence Data Collections

The workflow was tested using two large collections of data sets arising from human [45,59] and mice experiments ([7]; https://mothur.org/ accessed on 30 April 2021). In short, a multi-disease hospitalized cohort included various gastroenterological pathologies: ulcerative colitis, Crohn’s disease, tumor, infection, cirrhosis and peptic ulcer, unidentifiable abdominal pain. Gastrointestinal patients (*n* = 121) from a single ward were compared to 186 healthy volunteers [45] in order to fine-map the gut microbiota dysbiosis, using the bacterial (V3 V4) amplicon sequencing. In total, 6.6 million pairs of sequences were analyzed with an average coverage of 35,484 pairs of sequence reads from the 16S rRNA gene.

The mice dataset explored the separation between daily murine fecal samples (*n* = 360) obtained from C57BL/6 male and female mice at 0 to 9 (early) and 141 to 150 (late) days after weaning [7]. In total, 4.3 million pairs of sequence reads from the 16S rRNA gene with an average coverage of 9913 pairs of V4V5 reads per sample [22] were analyzed. During the first 150 days post weaning mice were allowed ad libitum feed with no specific influence in order to monitor whether the rapid change in weight at 10 days post weaning (obesity) affected the stability microbiome compared to the microbiome observed between days 140 and 150.

### 3.3. Statistical Analyses and Machine Learning

The two 16S rRNA sequence data collections were analyzed using GUMPP and according to three layers of information, namely genus, 97% OTU and ASV, and the additional three data types were calculated using PICRUSt2 integrated in GUMPP: predicted metagenomes; enzyme reactions; metabolic pathways. Piphillin-ready outputs for clinical exploration were calculated alongside, formatted and prepared. The underlying settings used in these analyses are part of the GUMPP configuration file and can be utilized and shared among researchers for reproducibility and ease of additional calculations. The resulting genus level data analysis of human gut microbiomes (four data matrices (16S rRNA; metagenomes; enzyme reactions; metabolic pathways) were subjected to machine learning in JADBIO [47] (version 1.1.164) for identification of microbial, genetic, enzymatic and pathway variables responsible for separation of the healthy and patient groups.

JADBIO [47] provides high-quality predictive models for diagnostics using state-of-the-art statistical and machine learning methods. Personal analytical biases and methodological statistical errors were eliminated from the analysis by autonomous exploration of several settings in modeling steps, exploring wide analytical space and producing convincing discovered features to discriminate between patients and healthy individuals. The JADBIO approach was adopted for modeling because of number of reason: First, automated parameter and algorithm selection without human inference enables testing and coverage of a wide machine learning algorithm-settings space. Second, JADBIO includes several algorithms for feature selection and modeling (linear regression, SVM, decision trees, random forest and Gaussian kernel SVMs) and all possible options with different parameters are tested during the process. Third, the obtained models were trained with different configurations of sub-data of the original dataset (all results are cross-validated with recently developed Bootstrap Bias Corrected CV (BBC-CV) [61]). Fourth, analyses were run on data with biomedical characteristics (sparse matrices, nonnormal distributions). Algorithm, hyperparameter and space selection protocols (AHPS) in JADBIO were used for selecting the most appropriate algorithm for preprocessing and transformation of a given dataset, for feature selection and modeling. The output of AHPS step was then evaluated through the configuration evaluation protocol in order to find the optimal model configuration for a given dataset [46,47]. JADBIO 1.1.164 was used with extensive tuning effort and 6 CPU cores in modelling various dataset selections. All four datasets were split 70 to 30 according to machine learning protocols. The training set (70% of the data in the dataset) was used to build the best interpretable models and the rest of the data (30%) was used for performance validations at all four levels of data analysis (16S rRNA genus level (424 features), KO (6126 features), EC (1887 features), pathways (365 features)). The area under the curve (AUC) metric was used to evaluate model performance. In total, the analytical space of algorithms and their corresponding settings was explored and 5960 of models and their individual settings were tested for genus and 11,920 for functional gene, enzymatic reactions and metabolic pathways, before the optimal configuration for the most informative model were obtained.

In addition to this, statistical power analysis of human microbiome data was performed [45,59] on all four data levels: 16S rRNA, KO, EC and pathways, between patients with different diseases and healthy individuals and according to presence/absence of the disease. Data was cube root normalized and mean centered. False discovery rate set to 0.1 was used in MetaboAnalyst module prepared for data analysis of population and metabolic studies [62].

All models created in analyses of the human gastrointestinal dataset can also be run on the local machine and are provided as part of the supplementary data (for local model execution, see the instructions in the electronic supplementary materials).

Mice data (*n* = 365) were processed and analyzed as described above in order to explore the differences between the four data types (16S rRNA; metagenomes; enzyme reactions; metabolic pathways) in terms of consistency of intersample relationships between the three layers of information routinely utilized in studies (genus; OTU; ASV). The intersample relationships were assessed by Mantel tests (*p* < 0.0002) utilizing (i) Pearson and (ii) Spearman correlation between data matrices (Bray-Curtis distance measure) and permutations (*n* = 9999) in either vegan-R [63] and/or PAST software (version 2.17c) [64]. The Mantel test tests the correlation between two distance matrices. It is non-parametric test and computes the significance of the correlation through permutations of the rows and columns of the input distance matrices.

## 4. Conclusions

By including the user preferences of genus, OTU or ASV type of analyses, GUMPP is the first workflow that introduces traceability and portability of all its parameters used in analyses. The workflow integrates and orchestrates end to end the inputs and outputs of the highly cited programs Mothur, PICRUSt2 and Pipihillin, controlled by Python code, delivered as portable Singularity image and accompanied by customizable configuration files. The whole GUMPP workflow can be executed for teaching or/and research purposes using personal computer or HPC. The ability to support reproducible analyses enables production of datasets that match multiomics layers of information, such as metagenomics, metaproteomics and metabolomics that ultimately identify the biochemical pathways characteristic of certain pathology [8]. These datasets coupled to biostatistics and mathematical approaches of machine learning can play significant role in extraction of truly significant and meaningful information from wide array of previously unexplored datasets (e.g., [45,59]) in relation to (i) a number of diseases (metabolic [65] or neurodegenerative [66] diseases), (ii) medical interventions, manipulations of bacteria-gut-brain axis [67] or (iii) treatment strategies for complex diseases [68]. The adoption of GUMPP in the gut-microbiota related research enables focusing on the identification of novel biomarkers that can lead to the development of mechanistic hypotheses applicable to the development of novel therapies in personalized medicine [2,9].

## Data Availability

GUMPP Singularity image, next to accompanying config files, manual and demo data is available here for download: http://gumpp.fe.uni-lj.si. The calculated models are made available as Electronic Supplementary Materials. Human and mice sequencing data were previously published and are available from original publications ([45,59] and ([7]; https://mothur.org/), respectively).

## Supplementary Materials

The following are available online at https://www.mdpi.com/article/10.3390/metabo11060336/s1. Figure S1: A schematic overview of data layers, Figure S2: The data can be analyzed at three different levels, Figure S3: An overview of the modelling step based on the four layers of information obtained through the use of GUMPP, Figure S4: An overview of characteristics of the models based on 16S rRNA, predicted metagenomes (KO), predicted enzymatic reactions (EC) and metabolic pathways (Pathway) data. KO and EC data performed slightly better than those based on 16S rRNA and pathway data, Table S1: Performance metrics of built models based on four different levels of data generated by GUMPP from human dataset, Table S2: Human dataset, power analysis. Sample size corresponding to calculated statistical power, Minimanual 1: GUMPP’s quick run routine, Minimanual 2: Instructions for running a model on a local machine.
