# Supplementary material for: General Unified Microbiome Profiling Pipeline (GUMPP) for Large Scale, Streamlined and Reproducible Analysis of Bacterial 16S rRNA Data to Predicted Microbial Metagenomes, Enzymatic Reactions and Metabolic Pathways
Source: Metabolites. 2021 May 24;11(6):336. doi: 10.3390/metabo11060336 (PMC8225202; doi:10.3390/metabo11060336)
Supplement: Supplementary file 1 [file metabolites-11-00336-s001.zip › metabolites-1197562-SI.pdf]

Electronic supplementary material

General Unified Microbiome Profiling Pipeline (GUMPP) for large scale, streamlined and reproducible analysis of bacterial 16S rRNA data to predicted microbial metagenomes, enzymatic reactions and metabolic pathways

Boštjan Murovec[1], Leon Deutsch[2], Blaž Stres[2,3,4,5]

[1] University of Ljubljana, Faculty of Electrical Engineering, Tržaška 25, SI-1000 Ljubljana, Slovenia

[2] University of Ljubljana, Biotechnical Faculty, Jamnikarjeva 101, SI-1000 Ljubljana, Slovenia

[3] University of Ljubljana, Faculty of Civil and Geodetic Engineering, Jamova 2, SI-1000 Ljubljana, Slovenia

[4] Jožef Stefan Institute, Department of Automation, Biocybernetics and Robotics, Jamova 39, SI-1000 Ljubljana, Slovenia

[5] University of Innsbruck, Department of Microbiology, Technikerstrasse 25d, A-6020 Innsbruck, Austria

Correspondence to Blaz Stres, University of Ljubljana, Biotechnical Faculty, Group for Microbiology and Microbial Biotechnology, Jamnikarjeva 101, 1000 Ljubljana, Slovenia. Email: blaz.stres@bf.uni-lj.si; Phone: +38641567633, Fax: +386 1 724 10 05.

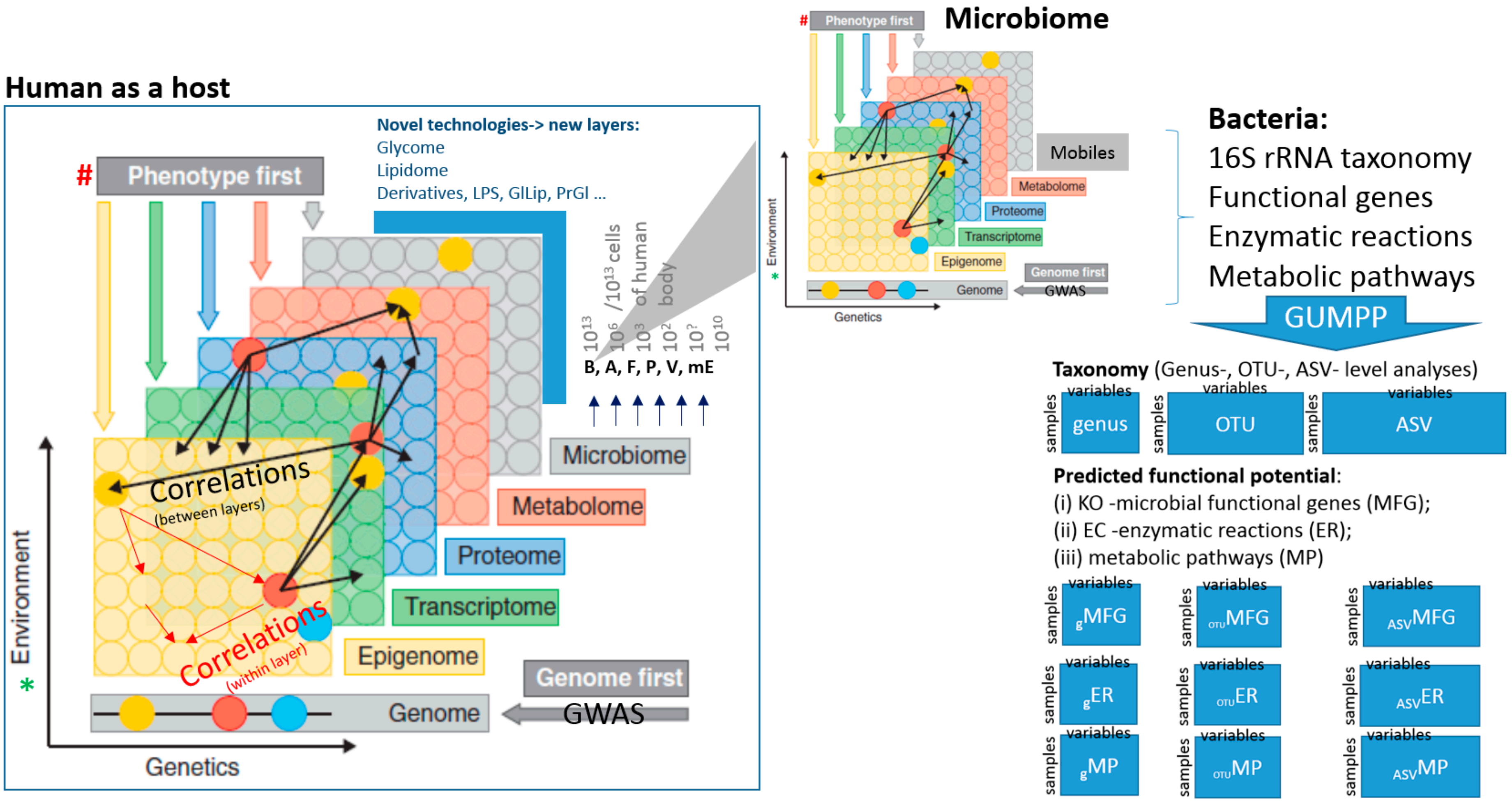

Figure S1: A schematic overview of data layers reported in published literature as parts of data driven approach to human disease exploration (Stres and Kronegger, 2019) (CC-BY Licence). The various data matrices generated using the GUMPP are presented in more detail in Figure S2. The data can be analyzed at genus, OTU- and/or ASV- levels. OTU- Operational Taxonomic Units (generally 97% identity of 16S rRNA); ASV – Amplicon Sequence Variants (unique sequence variants). KO – KEGG Orthologs (Kyoto Encyclopedia of Genes and Genomes); EC - Enzyme Commission number; For each level, four output tables are generated (e.g. genus, gMFG, gER, gMP). This figure serves as an example of the multilevel and multi-omic layers of information that are being

integrated in current multi-scale approaches to the integration of microbiological information into natural systems. Circles represent the entire pool of molecules detected in various 'omic' data layers. Genetic regulations and environments are embedded within all data layers, except the genome (GWAS) layer, and can affect each individual molecule to a different extent. The potential interactions or correlations between molecules detected within one layer or between different layers are represented by thin red and black arrows, respectively. As an example of the conceptual framework for consolidating multi-omic data and to understand the function of the system, the gene in a genome (blue circle) is epigenetically regulated (red circle) and controls multiple transcription targets correlated with multiple proteins that generate metabolites, which can have a greater influence on the microbiome layer as well. The three firsts (i.e. the genome first, the phenotype first and the environment first) imply a starting point: the associated locus versus any other layer versus environmental perturbations (i.e. thermodynamic boundaries within which the system routinely operates). GWAS: genome-wide association studies; B: bacteria; A: archaea; F: fungi; P: protozoa; V: viruses; mE: mobile elements; LPS - Lipopolysaccharides; GlLip - Glycolipids; PrGl – proteoglycans (Stres and Kronegger, 2019).

Taxonomy (Genus-, OTU-, ASV- level analyses)

Predicted functional potential analyses:
(i) KO -microbial functional genes (MFG);

(ii) EC -enzymatic reactions (ER);

(iii) metabolic pathways (MP)

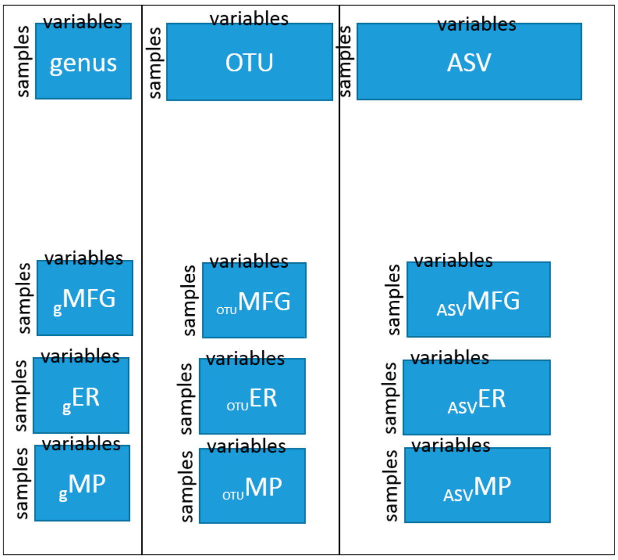

Figure S2. The data can be analyzed at genus-, OTU- and/or ASV- levels. The resulting three blocks containing each four data matrices describing specific information (taxonomy, functional genes, enzymatic reactions, metabolic pathways) can be seen. These data can be used for specific downstream data integration. The in depth description of the results of such data integration was beyond the scope of this study. OTU- Operational Taxonomic Units (generally 97% identity of 16S rRNA); ASV – Amplicon Sequence Variants (unique sequence variants). KO – KEGG Orthologs (Kyoto Encyclopedia of Genes and Genomes Orthologs); EC - Enzyme Commission number. Hence, for each level, four output tables are generated for further statistical analyses.

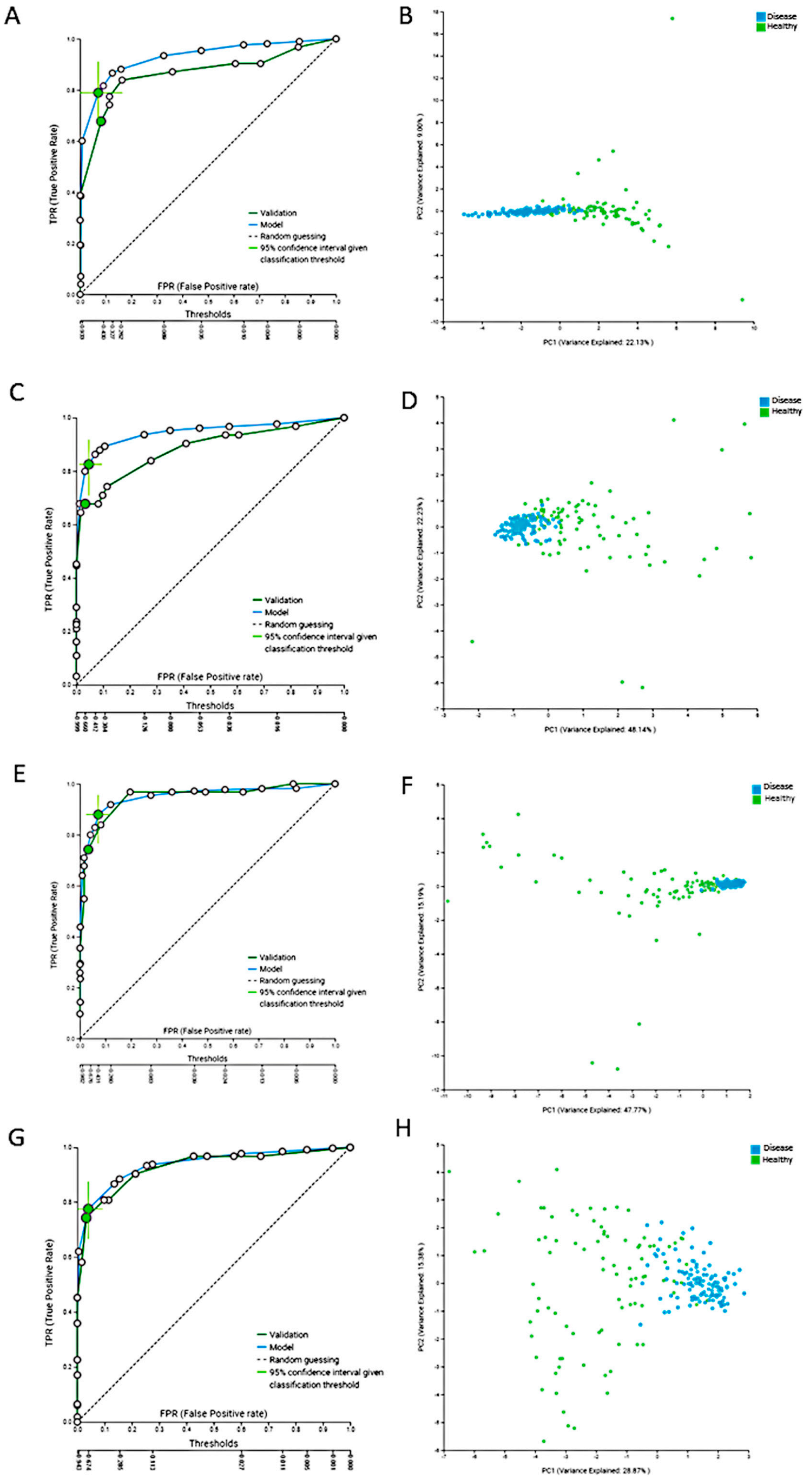
A
TPR (True Positive Rate)
FPR (False Positive rate)
Thresholds
Validation
Model
Random guessing
95% confidence interval given classification threshold
B
Disease
Healthy
PC1 (Variance Explained: 22.13%)
C
TPR (True Positive Rate)
FPR (False Positive rate)
Thresholds
Validation
Model
Random guessing
95% confidence interval given classification threshold
D
Disease
Healthy
PC1 (Variance Explained: 48.14%)
E
TPR (True Positive Rate)
FPR (False Positive rate)
Thresholds
Validation
Model
Random guessing
95% confidence interval given classification threshold
F
Disease
Healthy
PC1 (Variance Explained: 47.77%)
G
TPR (True Positive Rate)
FPR (False Positive rate)
Thresholds
Validation
Model
Random guessing
95% confidence interval given classification threshold
H
Disease
Healthy
PC1 (Variance Explained: 28.87%)

Figure S3. An overview of the modelling step based on the four layers of information obtained through the use of GUMPP. Receiver operating curves (A,C,E,G) representing model performance at all four levels: (A) 16S rRNA genus level taxonomy, (C) KO – functional genes, (E) EC – enzymatic reactions, and (G) metabolic pathways. The blue line represents the model generation from the dataset and the green line shows the evaluation of the model on the test dataset. White circles represent thresholds for ROC curve and predictive performance of the model from the optimal specificity to optimal sensitivity. In analogy to the right (B,D,F,H) there are the corresponding PCA plots showing the differences between the healthy (green) and diseased (blue) groups.

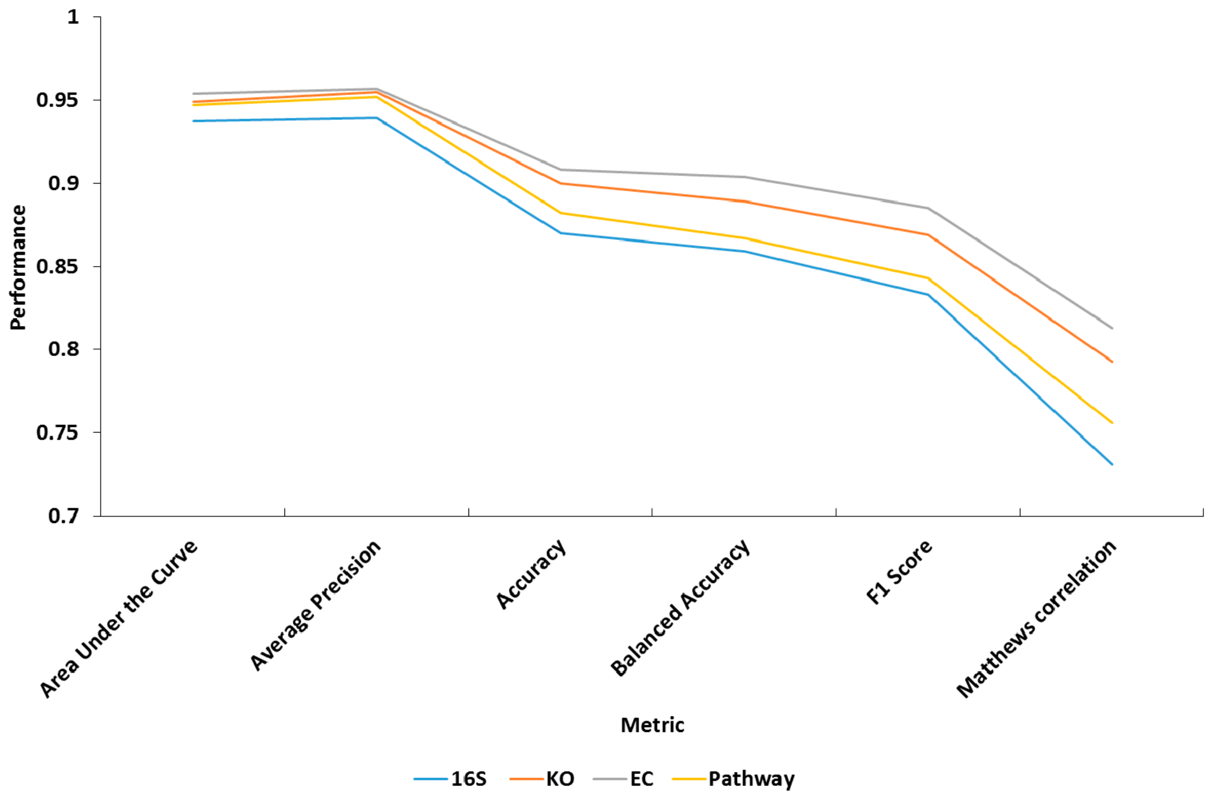

Figure S4. An overview of performance characteristics of the JADBIO models based on human intestinal tract data spanning the following data types: 16S rRNA, predicted metagenomes (KO), predicted enzymatic reactions (EC) and metabolic pathways (Pathway). KO and EC data performed slightly better than those based on 16S rRNA and pathway data. Please note the scale of Performance (y-axis).

Table S1. Performance metrics of built models based on four different levels of data generated by GUMPP from human dataset.

Metric	16S	KO	E.C.	Pathway
Area Under the Curve	0.937	0.949	0.954	0.947
Average Precision	0.939	0.955	0.957	0.952
Accuracy	0.87	0.9	0.908	0.882
Balanced Accuracy	0.859	0.889	0.904	0.867
F1 Score	0.833	0.869	0.885	0.843
Matthews correlation	0.731	0.793	0.813	0.756
Precision	0.894	0.931	0.906	0.94
True Positive Rate	0.789	0.825	0.879	0.774
Specificity	0.929	0.953	0.928	0.96
True Positives (TP)	0.33	0.346	0.369	0.324
True Negatives (TN)	0.041	0.027	0.539	0.023
False Positives (FP)	0.54	0.554	0.042	0.558
False Negatives (FN)	0.089	0.073	0.05	0.095
Average F1 Score	0.859	0.891	0.898	0.858
Average Matthews correlation	0.727	0.798	0.789	0.731

Table S2. Human dataset, power analysis. Sample size corresponding to calculated statistical power.

Pair	16S		KO		EC		Pathway	
	Stat. Power	Sample size	Stat. Power	Sample size	Stat. Power	Sample size	Stat. Power	Sample size
CD_Healthy	0.48	1000	0.81	6	0.8	60	0.82	40
CD_Infection	0.7	1000	1	1000	0.66	1000	0.66	1000
CD_Other	0.75	1000	0.74	1000	0.8	600	0.81	500
CD_tumor	0.8	500	0.8	150	0.8	200	0.8	200
CD_UC	0.69	1000	0.73	1000	0.69	1000	0.74	1000
Healthy _infection	0.55	1000	0.79	60	0.84	100	0.85	100
Healthy _other	0.55	1000	1	1000	0.8	100	0.84	40
Healthy _tumor	0.6	1000	0.83	150	0.8	400	0.81	300
Healthy _UC	0.8	1000	0.86	100	0.8	100	0.81	150
Infection_other	0.62	1000	0.8	1000	0.78	1000	0.8	1000
Infection_tumor	0.78	1000	0.7	1000	0.82	900	0.81	1000
Infection_UC	0.62	1000	0.58	1000	0.53	1000	0.57	1000
Other_tumor	0.63	1000	0.54	1000	0.49	1000	0.41	1000
Other_UC	0.7	1000	0.55	1000	0.57	1000	0.54	1000
Tumor_UC	0.82	1000	0.67	1000	0.79	1000	0.74	1000
Presence_absence	0.6	1000	1	10	0.86	60	0.86	40

Minimanual 1:

GUMPP's Quick Run Outline

Prerequisites

- Assure that Singularity version 3.x or 2.6.x is installed (https://sylabs.io). Install from repository of your Linux distribution, if needed.
- Assure that Squash file system (squashfs) is installed. Install from repository of your Linux distribution, if needed.
- Download GUMPP's singularity image (http://gumpp.fe.uni-lj.si) into a directory of your choice.

Preparation

- Create a directory (e.g. test) in your home directory and populate it with paired reads.
- Download configuration template http://gumpp.fe.uni-lj.si/config_template.txt into directory with input reads and give it a name (e.g. config.txt).
- According to instructions in config file set at least parameters: in_dir, msp_screen_seq_start, msp_screen_seq_end and msp_sub_sample_size.
- Set type of an analysis (if not ASV), and filtering of improper sequences according to their length by msp_screen_seq_min_length and msp_screen_seq_max_length.

```
# Config file example.
# Do NOT blindly apply the
# settings below, since they
# likely will not work for
# your inputs.

in_dir = /home/alice/samples

msp_screen_seq_start = 6388
msp_screen_seq_end = 25316

msp_sub_sample_size = 3000

msp_screen_seq_min_length = 430
msp_screen_seq_max_length = 465

analysis_asv=yes
#analysis_otu=yes
#analysis_gen=yes
```

Execution

- Start GUMPP by invoking the appropriate (suitably adopted) command line:

For Singularity 2.6.x:

```
singularity run /abs_path/gumpp_v1.simg
        /home/username/test/config.txt
```

For Singularity 3.x:

```
singularity run –writable-tmpfs /abs_path/gumpp_v1.simg
        /home/username/test/config.txt
```

```
-----------------------------------------------------
-----------------------------------------------------
Handling states and activities of executable units...
-----------------------------------------------------
-----------------------------------------------------


----------------------------------
  A. State evaluation pass 1:

     evaluating: results__PicRust2: generic_gen_original


--------------------------------
  Z. Applying execution policy:

     starting: results__PicRust2 generic_gen_original


===============================================================================
Summary of completed steps:
         input__orientation__orient_fasta duration 0:00:02
         results__piphillin_inputs duration 0:00:02
    Avg. input__Mothur_biom__Mothur_script duration 0:16:29 for 2 runs (min: 0:03:54, max: 0:29:05)
    Avg. input__gunzip duration 0:00:01 for 720 runs (min: 0:00:00, max: 0:00:02)

_______________________________________________________________________________
Steps that are being executed (CPUs, disk %, memory GB, start time, tag):

results__PicRust2: 1
--------------------
 16.00    15.00          10    2021-04-07__13:33:14    generic_gen_original
===============================================================================
2021-04-07 13:33:14    duration: 0:37:07    (to be updated on state change)
```

Figure R1: An example of the GUMPP during processing.

```
===============================================================================
Summary of completed steps:
         input__orientation__orient_fasta duration 0:00:02
         results__PicRust2 duration 0:03:54
         results__piphillin_inputs duration 0:00:02
    Avg. input__Mothur_biom__Mothur_script duration 0:16:29 for 2 runs (min: 0:03:54, max: 0:29:05)
    Avg. input__gunzip duration 0:00:01 for 720 runs (min: 0:00:00, max: 0:00:02)

===============================================================================
2021-04-07 13:37:09    duration: 0:41:02    (to be updated on state change)



----------------------------------------------------------------------
Summary of activities
----------------------------------------------------------------------

No errors or warnings were encountered during workflow execution.

Saving summary to a report file ... Finished.

Total duration: 0:41:02
```

Figure R2: An example of the GUMPP at final report of the successful run.

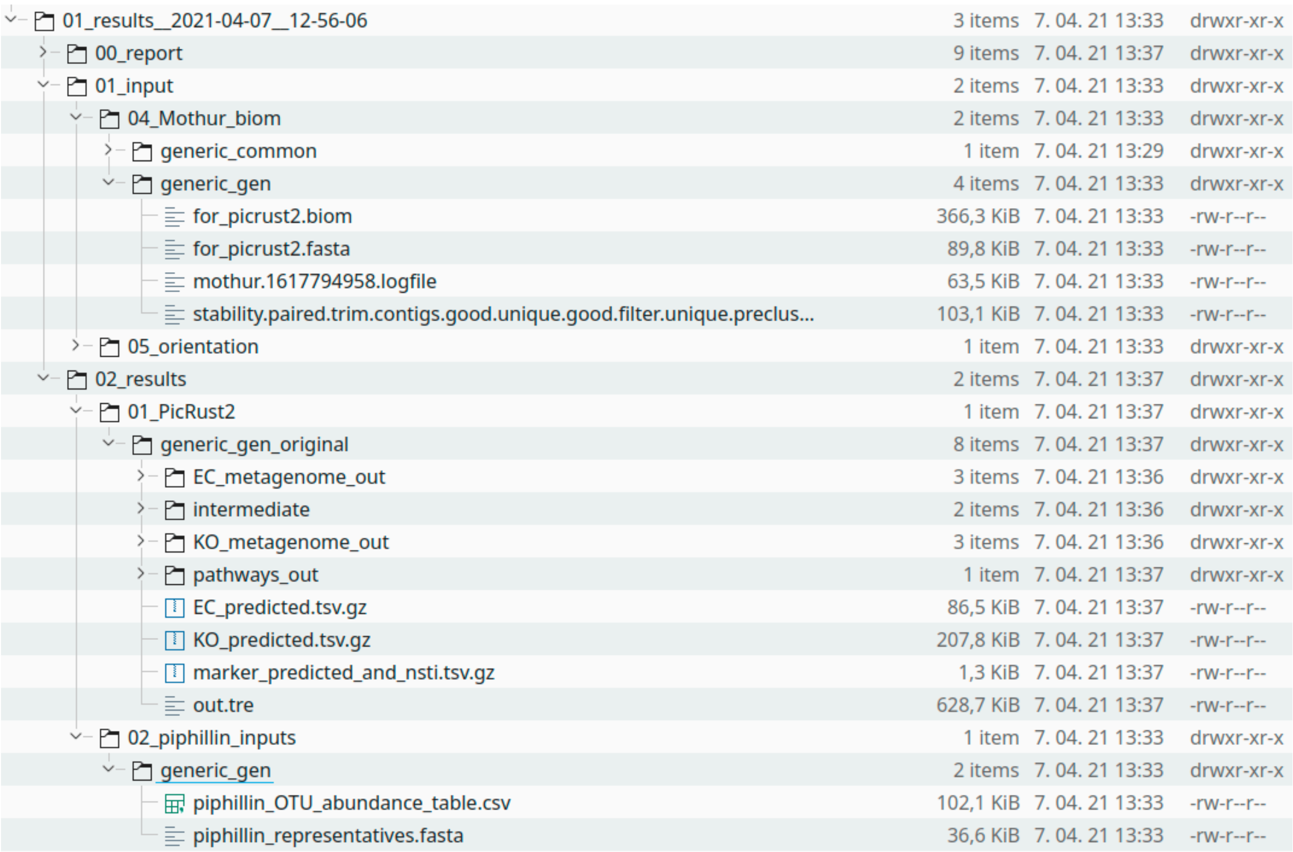

Figure R3: An example of the GUMPP output files and directory hierarchy produced after the successful run.

Minimanual 2: Instructions for running a model on a local machine

JADBIO allows the user to download a model and run it on a local machine.

To run our model locally, the user must meet the following requirements:

1. Java SE Development Kit version 15 (https://www.oracle.com/java/technologies/javase-jdk15-downloads.html)

2. the Java executor (contained in model.zip - filename: jadbio-1.1.182-model-exe.jar)

3. model (contained in model.zip - filename: jadbio-1.1.164-model.<datasetname>.bin)

After installing Java SE JDK, modelGUMPP.zip must be saved somewhere on the local machine. After saving model.zip, provided by the authors, the folder must be extracted (e.g. with WinZip, 7zip). The model must be executed with the command prompt (cmd) (Fig. 1, 2).

Step 1

Using the `cd path` command (Fig. 1), the user navigates to the same directory (e.g. Folder) that contains the model executor (.jar) and the model (.bin). In our case, the folder was located on desktop.

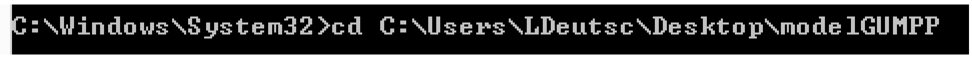

Figure I1. First command to navigate to the folder containing the model. In this case, we used the `cd C:\Users\LDeutsc\Desktop\model` command because Executor and Model were in the Model folder on the desktop.

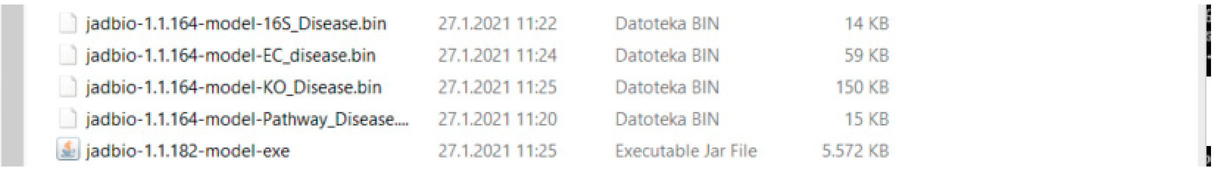

Figure I2. Files needed for the overview of the models created on JADBIO platform. All files are contained in the modelGUMPP.zip folder.

Step 2

The next step is to preview the model using the following command:

```
Java --enable-preview -jar jadbio-1.1.182-model-exe.jar -n jadbio-1.1.164-model-16S_Disease.bin
```

This allows the user to get an overview of the model, key features and information about the analysis (which algorithm was used, version of JADBIO and other information about the model) (Fig. 3). In upper case, model for 16S dataset was previewed. With the same approach, user can preview all other levels of information (KO, EC, Pathway). All model are part of modelGUMPP.zip file.

```
C:\Users\LDeutsc\Desktop\modelGUMPP>Java --enable-preview -jar jadbio-1.1.182-mo
del-exe.jar -n jadbio-1.1.164-model-16S_Disease.bin

Model created by JAD version:
  1.1.164

Model:
  Ridge Logistic Regression Model

Signature variables:
  Otu016,Otu028,Otu077,Otu019,Otu010,Otu005,Otu112,Otu004,Otu051,Otu079,Otu036,O
tu053,Otu026,Otu212,Otu151,Otu020,Otu021,Otu130,Otu113,Otu002,Otu207,Otu048,Otu0
43,Otu012,Otu066

Analysis info:
  id=10993, title='Otu_Disease', type='CLASSIFICATION', target='Disease', datase
t='Training_Otu', dataset_id='10699', project='MB_PresenceOfDisease', metric='AU
C', feature_selection=true, interpretable=false, max_vars=25, max_signatures=nul
l
```

Figure I3. Overview of the model.

Step 3

To test the model, the user must prepare data. The user needs to prepare the dataset as shown in Figure 4 and save it in a .csv document (comma separated values). After preparation, the prepared dataset must be saved in the same directory as the model executor and the model itself. Already prepared datasets are included into modelGUMPP.zip folder. Samples from patients are marked with Gxxx and samples from healthy individuals are marked with ZPxxx.

Sample	Otu001	Otu002	Otu003	Otu004	Otu005	Otu006	Otu007	Otu008	Otu009	Otu010	Otu011	Otu012
G002	249.5	79	0	0	195.67	0	0	0	0	0	4.88	0
G003	32.33	35	162	10	11.33	441	0	259	2.62	48	0.12	0.5
G006	82	53	107	79	6.67	129	0.2	72	5.5	123	0	0
G007	151.83	55	331.5	100	130	23	0	28	0.88	20	0.5	0
G008	107	36	36	5	2	60	11	5	7.38	0	0	0
G009	216	226	138	0	186	0	0	0	0.25	0	0.5	1
G010	147.17	139	68	124	0	59	0	122	10.62	144	0.62	0
G011	243	340	175	141	7.67	50	0	27	3	1	0	0
G013	0	0	0	0	954.67	0	0	0	0	0	2.25	0
G015	0.5	0	1	0	0.33	0	0	0	0	0	0	1296
G016	62.5	71	153	104	0	19	156	23	25.62	24	1.25	0.5
G017	75.67	32	113	78	0	91	0	306	8.12	9	0	0
G018	0.17	1	0	1	0	0	0	1	0	0	0.12	1230.5
G019	134.17	85	0	289	8	173	0	263	37.88	0	0	5.5
G021	66.67	100	106.5	95	0.67	2	207.4	11	3.5	9	0.12	0
G022	148.5	70	16.5	153	337.67	36	0.4	0	1.12	14	0.5	23
G023	50.33	167	30	60	10.33	64	9.2	85	40.25	67	0	0.5
G024	317	36	42.5	147	0	2	0.2	0	36.88	0	0	2.5
G025	81.83	87	168.5	119	3	84	0	14	5.38	159	4.88	10.5
G026	0.83	0	0	0	0	0	0	0	0	0	59.75	1126
G027	107	446	0.5	9	27.33	15	0.2	1	0.25	0	0.12	0.5
G029	54	61	57.5	83	0	1	0	10	0.75	1	0	878.5
ZP0201	130.5	204	191	83	0	116	0	5	3.88	192	0	0
ZP0202	85.33	429	221.5	61	0	74	0	2	7.12	111	0	0
ZP0203	116.17	192	200.5	139	3	91	0	4	7.38	41	0	0

Sample	Otu013	Otu014	Otu015	Otu016	Otu017	Otu018	Otu019	Otu020	Otu021	Otu022	Otu023
G002	531	0	0	6	0	0	0	0	0	0	0
G003	34	59	27	1.6	0	0	14	0	9	2	51
G006	39	178	174	1.6	1	28	47	7	36	33	37
G007	142	157	15	1	0	0.33	35	10	13	3	9
G008	131	1	2	2.4	2	0	0	0	3	1	1.5
G009	0	0	0	9.2	0	0	0	0	45	0	0
G010	0	221	47	0.6	0	15.33	27	25	2	7	10.5
G011	0	13	22	0	0	0	18	90	22	66	0
G013	0	0	0	14.8	5	0	0	0	0	0	0
G015	0	0	0	22.6	175	0	0	0	0	0	0
G016	66	37	15	0.2	0	0	26	60	14	12	7
G017	38	65	32	12	640	111.33	35	26	46	13	7
G018	0	1	0	3.6	438	0	1	0	0	1	0
G019	47	0	0	0	0	162	0	0	16	0	26.5
G021	7	56	0	1	0	0	16	56	15	43	1
G022	3	41	10	10.8	1	0.67	18	42	2	10	4
G023	60	97	42	2.4	1	8	31	2	6	14	8.5
G024	0	0	0	0.6	0	0	0	0	0	0	0
G025	9	91	52	23.2	118	0	22	118	24	18	1
G026	1	0	0	2.8	23	0	0	0	0	0	0
G027	0	1	0	3	0	145	11	0	0	0	0.5
G029	5	11	0	0.6	4	0	3	16	19	18	0
ZP0201	27	96	80	0	0	13	36	33	20	122	0
ZP0202	18	144	88	5.4	6	5.33	30	94	36	28	0.5
ZP0203	0	223	69	0	0	0.67	53	12	48	30	3

Figure I4. Prepared dataset (Gxxx – patients, ZP – healthy individuals)

Step 4

After saving the dataset, the user must use the next command in the terminal:

```
java --enable-preview -jar jadbio-1.1.182-model-exe.jar -m jadbio-1.1.164-model-16S_Disease.bin -i 16S.csv -o 16S-output.csv
```

This command runs the model on test data (16S.csv in our case) and creates a new dataset with predictions (16S-output.csv) in the same directory (Fig. 5).

```
C:\Users\LDeutsc\Desktop\modelGUMPP>java --enable-preview -jar jadbio-1.1.182-model-exe.jar -m jadbio-1.1.164-model-16S_Disease.bin -i 16S.csv -o 16S-output.csv

Successfully loaded model from jadbio-1.1.164-model-16S_Disease.bin
Successfully loaded input dataset from 16S.csv
Successfully wrote predictions to 16S-output.csv
```

Figure I5. Executing the model and creating the output .csv file with predictions in the same directory.

Step 5

After model execution, the user can check the calculated predictions by opening the .csv file directly by clicking on the created .csv file and opening it in any data analysis program (Excel, Past, R ...). As shown in Fig. 6 the model correctly classifies the data as healthy and as disease. The first column is the same as in the test data created by the user. The second and third columns show the calculated probabilities for the Disease class (second column) and for the Healthy class (third column).

	A
1	Sample name,Prob (class = No),Prob (class = Yes)
2	G002,0.001002513998170461,0.9989974860018296
3	G003,0.006188487994796388,0.9938115120052037
4	G006,0.03244377167576495,0.9675562283242352
5	G007,0.21344073241469658,0.7865592675853034
6	G008,1.662205223675324E-14,0.9999999999999833
7	G009,0.00978294979197632,0.9902170502080238
8	G010,0.38059378538303423,0.6194062146169659
9	G011,0.3558986812911365,0.6441013187088634
10	G013,1.426042293669462E-6,0.9999985739577063
11	G015,1.0312013119790439E-4,0.9998968798688022
12	G016,0.6630697664911461,0.336930233508854
13	G017,0.7203809247765964,0.27961907522340357
14	G018,0.0021416344281555057,0.9978583655718445
15	G019,0.09418276552485502,0.905817234475145
16	G021,0.20145221019476015,0.7985477898052399
17	G022,0.023989052217264092,0.9760109477827359
18	G023,0.6566292779488401,0.34337072205116
19	G024,0.0036532571304570684,0.996346742869543
20	G025,0.20238492540629072,0.7976150745937093
21	G026,0.0020644605528634444,0.9979355394471365
22	G027,0.06767381891391504,0.932326181086085
23	G029,0.0169628259684254,0.9830371740315745
24	ZP0201,0.995606622909608,0.0043933770903919955
25	ZP0202,0.9844431548452719,0.015556845154728171
26	ZP0203,0.9713935861376919,0.02860641386230807
27	ZP0204,0.9628519972995334,0.03714800270046665
28	ZP0205,0.9964513828129297,0.0035486171870703262
29	ZP0206,0.9914327417839056,0.008567258216094274
30	ZP0207,0.9993187773316771,6.812226683229878E-4
31	ZP0208,0.9999922191682509,7.780831749115737E-6
32	ZP0209,0.9537423817282127,0.04625761827178735
33	ZP0210,0.992287910662175,0.007712089337825074 4
34	ZP0212,0.9997751800877602,2.2481991223975028E-4
35	ZP0213,0.9978587450615696,0.002141254938430459 6
36	ZP0215,0.9999771364612802,2.2863538719725003E-5
37	ZP0221,0.9999851754800285,1.4824519971509357E-5
38	ZP0223,0.9107685137747521,0.08923148622524796
39	ZP0224,0.9520707932608887,0.04792920673911125
40	ZP0226,0.8205028394788675,0.1794971605211325

Figure I6. The newly created .csv file with predictions calculated from the test data.
